# Supplementary figures and images for: Porphyromonas gingivalis induction of TLR2 association with Vinculin enables PI3K activation and immune evasion
Source: PLoS Pathog. 2023 Apr 6;19(4):e1011284. doi: 10.1371/journal.ppat.1011284 (PMC10112799; doi:10.1371/journal.ppat.1011284)

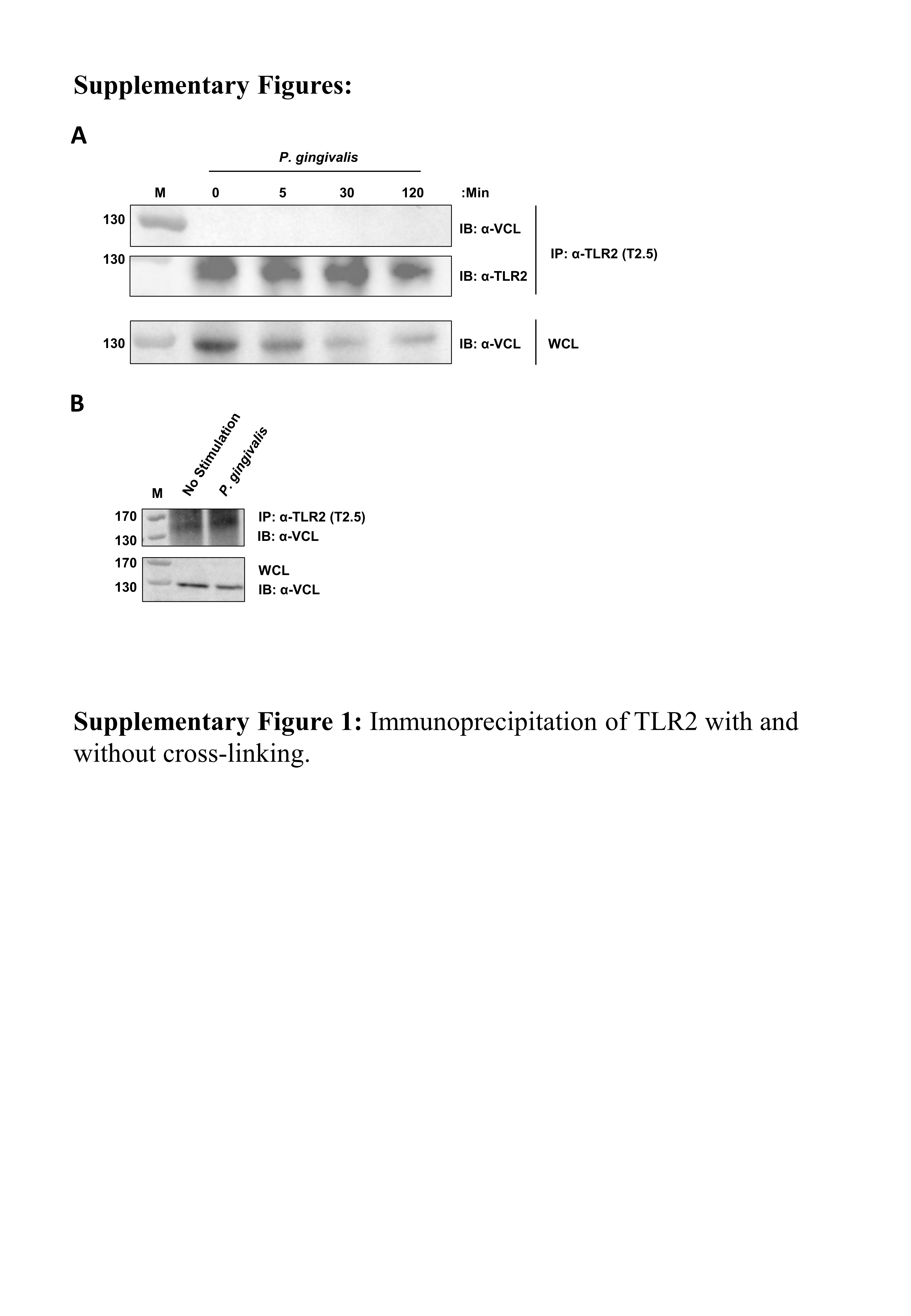

Supplement: S1 Fig — (A) Differentiated THP1 cells were stimulated with P. gingivalis (MOI 10) for the indicated times followed by immunoprecipitation of TLR2. Eluates were analyzed for VCL and TLR2, and whole cell lysates (WCL) were analyzed for VCL. (B) Differentiated PBMCs were infected with P. gingivalis (MOI 10) for 30 min followed by cross-linking with DSP (M, marker). TLR2 was immunoprecipitated (IP) and the boiled beads were analyzed for VCL by immunoblot (IB). Whole cell lysates (WCL) were analyzed to control for protein input and loading. (TIF) [file ppat.1011284.s001.TIF]

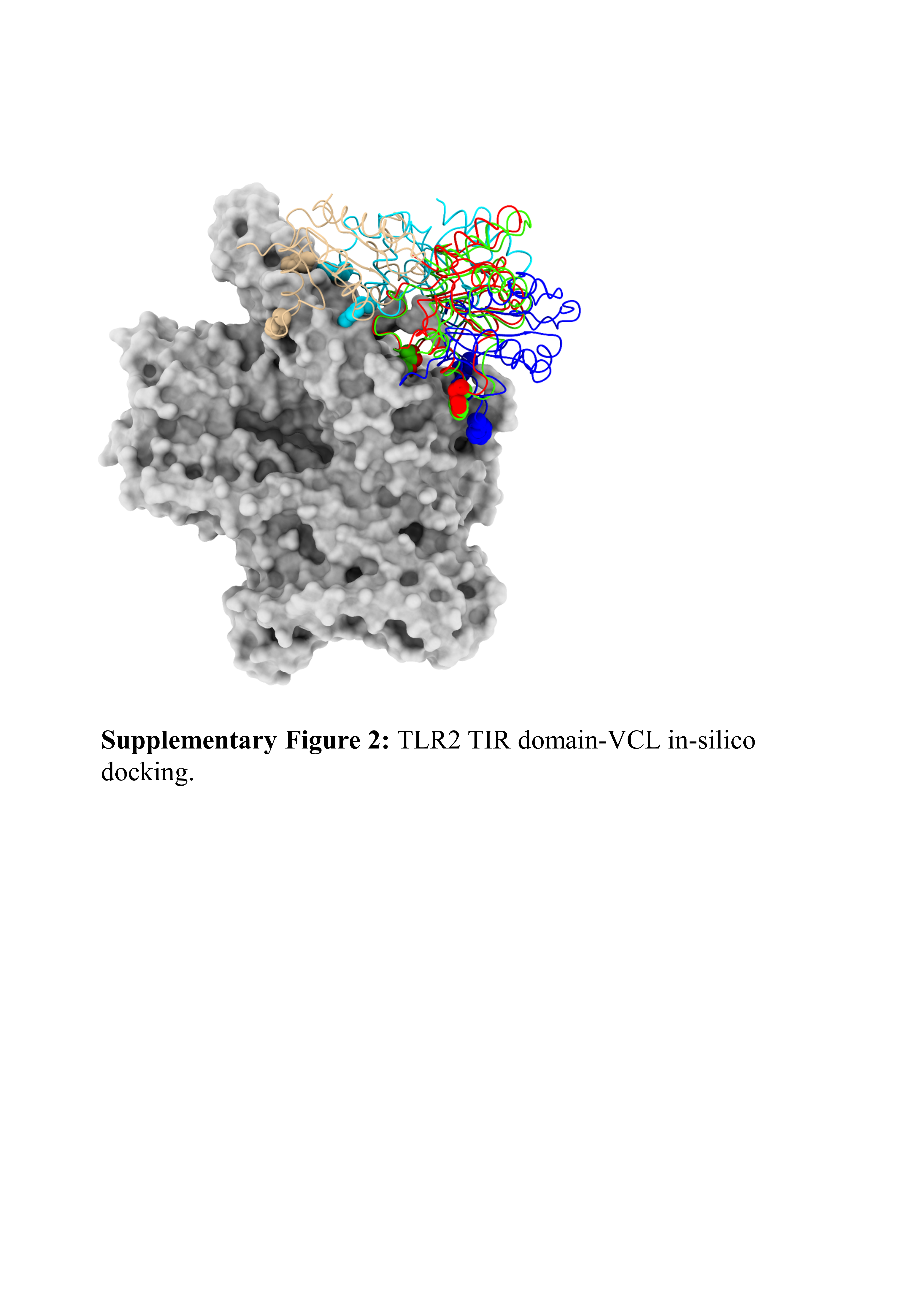

Supplement: S2 Fig — Five docking poses obtained from the pyDock docking simulation are shown in distinct colors. Residues W684 and F719 are shown in space-filling model. (TIF) [file ppat.1011284.s002.TIF]

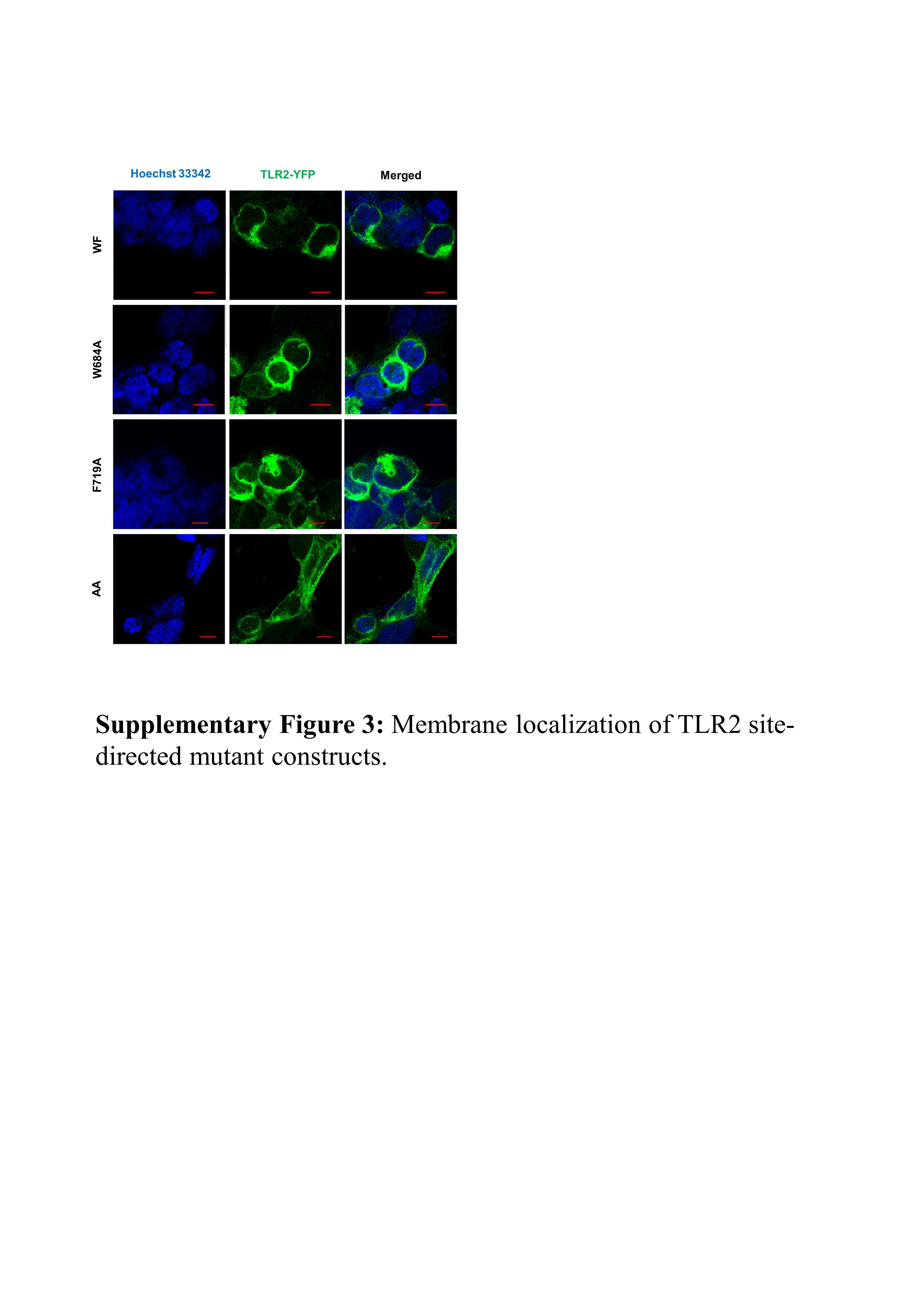

Supplement: S3 Fig — HEK293 cells were transiently transfected with native TLR2 (WF) and mutants W684A, F719A and AA-TLR2-YFP (green) and cells were fixed and nuclei were stained with Hoechst (blue). Images were captured using a NIKON confocal microscope at 60X magnification. (TIF) [file ppat.1011284.s003.TIF]

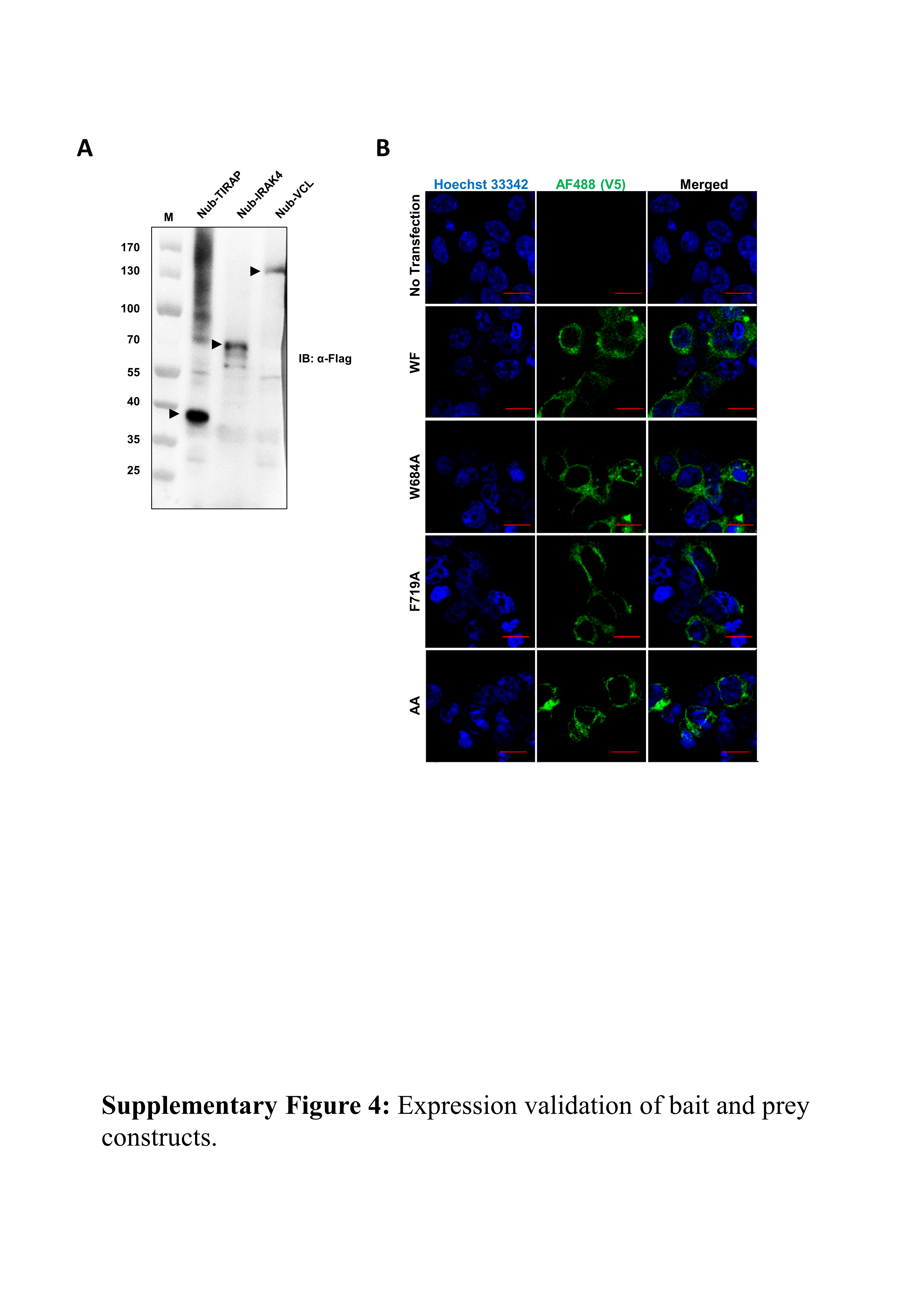

Supplement: S4 Fig — (A) MaMTH-modified HEK293T cells were transfected with respective Nub-prey vectors, and lysed after 48 h. Western blot shows the expression of prey constructs detected using anti-flag antibody. (B) HEK293 cells were transiently transfected with TLR2-Cub-V5 and mutants W684A, F719A and AA-TLR2-Cub-V5 (green) and cells were fixed and stained for nuclei with Hoechst (blue). Images were captured using a NIKON confocal microscope at 40X magnification. (TIF) [file ppat.1011284.s004.TIF]
