# Supplementary material for: Porphyromonas gingivalis induction of TLR2 association with Vinculin enables PI3K activation and immune evasion
Source: PLoS Pathog. 2023 Apr 6;19(4):e1011284. doi: 10.1371/journal.ppat.1011284 (PMC10112799; doi:10.1371/journal.ppat.1011284)
Supplement: Attachment — Submitted filename: Response to review_Pandi et al.pdf [file ppat.1011284.s007.pdf]

January 29, 2023

Dear Editors and Reviewers,

We greatly appreciate the thorough and careful review of our manuscript and the important corrections and comments. We have addressed the points raised below in a “point-by-point” response letter, and hope that the revised manuscript is acceptable for publication.

Sincerely yours,

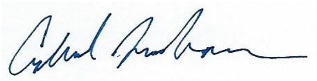

Gabriel Nussbaum (for the authors)

Reviewer #1: The manuscript is very well written and the pleasure to read. The only critical comments I have are pertinent to using the human macrophage cell line without verification of key results on primary cells. Therefore, I’m asking the authors to provide arguments for not doing that both in the rebuttal letter and in the discussion.

Response: New supplementary figure 1B shows the capture of vinculin following immunoprecipitation of TLR2 in primary human macrophages. In the response to reviewer #3 below, point #3, we explain further. The revised manuscript includes a discussion of this issue (p. 14, lines 299-302, lines 414-21).

Reviewer #2: (No Response)

Reviewer #3: 1- (Results lines 88-118). The authors indicate the TLR2 was pulled down in the DSP studies. It is not clear if TLR1 or TLR6 peptides were detected. In a similar light, it does not appear that TLR adaptor molecule peptides were detected either. Could the authors elaborate on this as it would be anticipated that one of these two TLRs would be present as TLR2 signals as a heterodimer as well as the absence of an associating adaptor molecule.

Response: We propose that limitations of the approach led to the inability to capture classical TLR2 partners such as TLR1, TLR6, TIRAP, and MyD88. A major limitation

(extrinsic to the methodology) is the fact that we only examined one time point of activation (30 min). The interactome will likely shift over time, and some time-points may contain more of the known TLR2 interacting proteins. A limitation that is intrinsic to the methodology is that membrane proteins, due to their hydrophobic characteristics and lipid-rich environment, require significant optimizations for detection by MS (new reference Schey et al., 2013). In addition to that, the amino acid compositions of some proteins obstruct the discovery of cross-linked residues, since the cross-linking reactions can alter the enzymatic digestion of protein peptides (new reference Dau et al., 2019). Of note, Kamal et al (PMID: 31221720) also utilized a cross-linking and immunoprecipitation followed by LC-MS approach (to study the effect of statins on TLR2 signaling induced by PAM) and did not recover known adapter proteins.

In the revised manuscript we include a full discussion of these limitations (p. 14).

2- (Results lines 88-118). In this same section, STRING analysis predicted that vinculin interaction with TLR2 is possible by is indirect via an interaction with paxlilin. The manuscript does a superb job exploring the TLR-vinculin interaction, but does not clarify experimentally the mechanism of direct vs indirect interaction – in other words is paxillin required for the TLR2-vinculin interaction to occur?

Response: We realize that the presentation of the STRING analysis adds confusion since it suggests a potential indirect association whereas our results all support a direct association of vinculin and TLR2. We had used the STRING analysis to help indicate which of the immunoprecipitated proteins have been shown to bear any relationship to TLR2. In fact, we did not find paxillin or SRC in the immunoprecipitated proteins, and since vinculin peptides were modified by the cross-linker we hypothesized, and experimentally pursued, a direct interaction with TLR2. In the revised manuscript we removed the referral to the STRING analysis – the papers supporting focal adhesion complex members such as paxillin associating with TLR2 (the basis for the STRING result) are referenced and discussed in the discussion.

3- The team does excellent work with THP-1 cells which are an established model cell used for exploring macrophage function. Is there any evidence that primary human macrophages recapitulate the findings with the THP-1 cells?

Response: We found that it is a significant technical challenge to immunoprecipitate native TLR2 from primary human macrophages (in fact, we are unable to find any references where TLR2 was immunoprecipitated from primary human macrophages). We are unable to visualize a strong band for immunoprecipitated TLR2 from primary macrophages (differentiated from PBMCs), in contrast to the THP1 cells. After cross-linking primary macrophages with DSP (the cross-linker used in our studies), and immunoprecipitation of TLR2, we do see vinculin, but only if we load the blot with more protein than usual, and although there is a difference between control and *P. gingivalis*-infected macrophages, the difference is not as pronounced as for the THP1 cells. A new supplementary figure showing this finding is included in the revised manuscript (Supplementary Figure 1B), and a statement of the limitation of the study is included in the revised discussion section (p. 14, lines 299-302, p. 19lines 414-21). Additions are also made to the methods and acknowledgements sections.

4- In Figure 5D. Was an irrelevant isotype-matched Ab used as a control?

Response: We had used the isotype control in experiments run in parallel to the presented experiments, and found that it did not have an effect on WT THP-1 macrophages. However, in recognition of the critique, we re-ran the experiments of Figure 5D using anti-TLR2 and its isotype control for the WT, shCntrol and shVCL macrophages, and replaced the figure with the new figure.

5- Statistical analyses. In some of the assessments made by the authors, there appears to be 3-way comparisons; however 2-way t-tests are indicated as the sole way data were analyzed. Unclear why ANOVA analyses were not considered?

Response: We re-analyzed the experiments that had multiple comparisons using ANOVA analysis. These analyses are incorporated to new figures 4D and 5D. In general, the significance of the outcome was strengthened in the new analysis. PAM signal in the split ubiquitin system, which was much lower than the signal induced by *P. gingivalis* stimulation and had more variance, shows a trend but is not significant in the new analysis (p. 24-25, lines 547-8).

Part III – Minor Issues: Editorial and Data Presentation Modifications
Please use this section for editorial suggestions as well as relatively minor modifications of existing data that would enhance clarity.

Reviewer #1: 1. Abbreviations are spelled out in the result section but not in the Methods section. This makes the method section difficult to comprehend if reading independently.

Response: In the revised version all abbreviations are spelled out in the methods section.

2. Page 17, lane 369: Apart from "1% 1M HEPES" it is appropriate to specify the final HEPES concentration (in mM). What was sodium pyruvate concentration?

Response: These corrections were made (p. 18, lines 395-6)

3. Page 18, lane 384: Is there a reference to statement that OD650 of 0.1 corresponds to 10^10^ CFU/mL?

Response: We added a reference to our original work where we determined this correspondence (p. 19, line 411-2).

4. Page 20, lane 430: at what MOI?

Response: The MOI was 10 and this information was added (p. 21, line 468).

Reviewer #2: 1. Line 59-60 – there are others receptors evolved to recognize P. gingivalis. They should be listed.

Response: We included reference to other receptors shown to be involved in the response to live *P. gingivalis* (new sentence plus references, p. 4 lines 59-62).

2. Line 97 – the corresponding Figure/Table should be indicated

Response: We made this correction (p. 6, lines 102-3)

3. Line 118 – lack of information about PAM stimulation (Fig. 1C)

Response: The information was added (p. 7, lines 117-18).

4. Line 153 – „HeLa cells, widely used for VCL studies …" – this sentence should appear already in the description of Fig. 1.

Response: We made this change (p. 7, lines 119).

5. Figure 3A – the control panel showing transfected cells without PAM stimulation should be presented.

Response: Figure 3A represents % induction over background luminescence of transfected cells not stimulated with PAM and therefore the non-stimulated cells are not shown. We clarified this in the figure y-axis label and in the legend (p. 31, lines 741-2).

6. Line 159 – „ …TLR2 co-localization was not significantly different …" – „ns" should be indicated in the Fig. 3B

Response: We added ns to the figure 3B (p. 31, line 748).

7. The lack of statistical analysis for PAM stimulation between mutants in the Fig. 4E. Moreover, the phenomenon was described for P. gingivalis but as PAM lipopeptide stimulation can lead to a similar effect (Fig. 4) the role of such signaling pathway for other pathogens should be discussed – at least the activation of PI3K by PAM and its immunomodulatory role.

Response: We added the statistical analyses to Figure 4. We also added a sentence to the discussion relating to the potential role of the pathway for other pathogens (p. 15, lines 310-13, p. 32, lines 761-2).

8. Fig. 4D – could be presented as supplementary data

Response: We agree and moved this figure to the supplementary data (supp. Figure 4B, p. 35, lines 831-4).

9. Line 251 – „P. gingivalis blocks phagolysosomal maturation and intracellular survival in macrophages…" – the sentence should be rephrased

Response: We corrected this mis-phrased sentence (thanks!) (p. 12, line 256)

10. As the authors showed diminished phagocytosis of P. gingivalis in VCL knock-

down macrophages (Fig. 5E) the percent of recovery could be calculated based on the comparison of engulfed bacteria (after 1 h of phagocytosis) to the number of CFU obtained after different time post infection. Those data could be presented as a separate panel. What is the MOI used in the analysis of bacterial survival? Was the analysis of bacterial recovery performed after longer time post infection (days)?

Response: We agree that we could normalize the intracellular survival to the number of engulfed bacteria and that would make the difference in intracellular survival even more pronounced. However, since we perform the intracellular survival assay using MOI 10 without fluorescent labeling of the bacteria, and the phagocytosis assay at MOI 100 using FITC-labeled bacteria we are unable to combine the data.

11. Figure 7 – what do the authors mean as steady state (1)? Non-infected cells? Then, how the improved killing of bacteria can be proposed?

Response: We made the necessary clarification to Figure 7 and its legend. We had meant un-infected macrophages and indeed the graphic did not make sense by showing survival. We think the legend and figure are now clear (p. 33, lines 800 & 802).

12. Line 506 – lack of description for „****"

Response: We added the description (p. 24-5, lines 547-8).

13. Line 657 – the figure legend should be supplemented by adding a description of **, ***, ns

Response: We added these descriptions (p. 30, lines 721-22).

14. Line 690 – the activation of TF was performed after 8h. Is it the optimal time for measurement of its activity?

Response: We tested 4 h, 6 h, and 8 h, and found that the signal was maximal at 8 h. We now added that information to the methods section. All repeat experiments for the figures were performed at 8 h time point (p. 24, lines 540-2).

15. Line 717 – lack of description for „****”

Response: Description added (p. 32, line 761-2).

16. Supplementary Table 1 – it would be valuable to present the data in a form that allows for the straight comparison of protein identified in resting and P. gingivalis-infected cells.

Response: We combined Table 1A and 1B to one table to enable this straight comparison (p. 36-37).

Reviewer #3: (No Response)
